# Medication Lists and Brown Bag Reviews: Potential Positive and Negative Impacts on Patients Beliefs about Their Medicine

**DOI:** 10.1155/2015/874067

**Published:** 2015-10-11

**Authors:** Cornelia Jäger, Jost Steinhaeuser, Tobias Freund, Joachim Szecsenyi, Katja Goetz

**Affiliations:** ^1^Department of General Practice and Health Services Research, University Hospital Heidelberg, Voßstraße 2, Geb. 37, 69115 Heidelberg, Germany; ^2^Institute of Family Medicine, University Hospital Schleswig-Holstein, Campus Lübeck, Ratzeburger Allee 160, Haus 50, 23538 Lübeck, Germany

## Abstract

*Introduction*. Medication lists and structured medication counselling (SMC) including “brown bag reviews” (BBR) are important instruments for medication safety. The aim of this study was to explore whether patients' use of a medication list is associated with their beliefs about their medicine and their memory of SMC.* Methods.* Baseline data of 344 patients enrolled into the “Polypharmacy in Multimorbid Patients study” were analysed. Linear regression models were calculated for the “specific necessity subscale” (SNS) and the “specific concerns subscale” (SCS) of the German “Beliefs About Medicine Questionnaire,” including self-developed variables assessing patients' use of a medication list, their memory of SMC, and sociodemographic data.* Results. *62.8% (*n* = 216) remembered an appointment for SMC and 32.0% (*n* = 110) BBR. The SNS correlated positively with regular receipt of a medication list (*β* = 0.286, *p* < 0.01) and negatively with memory of a BBR (*β* = −0.268; *p* < 0.01). The SCS correlated positively with memory of a BBR (*β* = 0.160, *p* = 0.02) and negatively with the comprehensiveness of the mediation list (*β* = −0.224; *p* < 0.01).* Conclusions. *A comprehensive medication list may reduce patients' concerns and increase the perceived necessity of their medication. A potential negative impact of BBR on patients' beliefs about their medicine should be considered and quality standards for SMC developed.

## 1. Introduction

As a consequence of demographic change and improved medical treatment, the number of patients with multiple chronic conditions and polypharmacy is constantly increasing [1]. These patients frequently require complex, interdisciplinary care involving multiple health care professionals and prescribers [2]. It is well known that an increasing number of prescribed drugs are associated with a higher risk of adverse drug reactions (ADR) [3] and hospitalisation [4, 5].

Medication errors are the most common preventable cause for these undesired events and comprise the prescribing, dispensing, and administration of the medicine [6]. Reasons leading to medication errors are manifold and also context-specific [7] but frequently involve patients' nonadherence [8] and suboptimal medication management, including the exchange about medication-related information among health care professionals [2].

Consequently, strengthening patients' self-management abilities concerning their medication and improving the exchange of medication-related data are important approaches to increasing medication safety [9]. In health care systems without established gate-keeping, such as Germany, this is particularly challenging, as patients do not have to be registered at any general practitioner and have free access to specialist care [10]. Furthermore, there is no established electronic system for data exchange between the different health care providers and settings in Germany [11]. To date, the printed, paper-based medication list is the most important document for medication-related information [12, 13]. However, deficits concerning the quality and availability of these medications lists are well known.

In Germany, 25–50% of patients with long-term medication have a medication list [14]. Several studies showed that discrepancies between the documented and actually taken medication appear in about 75% of the cases [15–17], of which 25% are considered potentially harmful [18]. Due to the lacking standardisation of the medication lists, important information is frequently lacking or—in case of handwritten medication lists—not readable [19].

An important instrument to increase the sufficiency and correctness of medication lists is a so-called “brown bag review,” an inventory of the medication actually taken by the patient based on the medication packages the patient is using [20]. According to a German guideline on multimedication [21], this review should be part of a specific appointment for “structured medication counselling” (SMC) at the general practitioner's clinic. During SMC an assessment of (undesired) effects of the medication and possible reasons for nonadherence, such as application problems or attitudes and concerns towards the medication, should be broached [22].

Within the “Polypharmacy in Multimorbid Patients Study (PomP)” a tailored intervention to implement SMC into primary care practices and to increase the quality and availability of medication lists has been developed and evaluated in a randomised controlled trial [23].

The aim of the current analysis was to explore whether patients' beliefs about their medicine are associated with the use of a medication list and the memory of medication counselling and brown bag review.

## 2. Methods

### 2.1. Participants

The study took place in the federal state Baden-Württemberg in Germany. Baseline data of all patients enrolled into the PomP study were analysed. Eligibility criteria for patients were assessed using insurance claim data and comprised

- age older than 50 years;
- multimorbidity, defined as diagnosis of at least three chronic conditions based on a previously published diagnosis list [24];
- polypharmacy, defined as repeated prescription of more than 4 drugs;
- enrolment in a special care contract of one large German health insurance (HZV AOK Baden-Wuerttemberg);
- high risk of medication problems according to the personal assessment of the general practitioner (GP), for example, nonadherence or previous hospitalisation due to medication related events.

### 2.2. Data Collection

The data were collected in October 2013. Patients completed questionnaires on an internet-based platform on a tablet PC in the practice of their treating general practitioner, after they had given written informed consent to participate in the PomP study. The data were stored on a secure central server of the University of Heidelberg.

The questionnaire included the specific part of the German version of the Beliefs in Medicines Questionnaire (BMQ-D) [25] as well as nonvalidated items on the presence and patients' use of medication lists, the memory of having ever received medication counselling and a brown bag review, and sociodemographic questions.

The BMQ-D has been validated and proved to be suitable to measure patients' beliefs in medicine in German primary care settings [26]. While the general part of the BMQ-D assesses the beliefs about medicines in general, the specific part used in this study focuses on patients' beliefs about the particular medication prescribed for them. It comprises two subscales. The “specific-necessity scale” (SNS) assesses patients' beliefs about their personal need for the medicine and how important the medicine is in maintaining their health now and in the future. The “specific-concerns scale” (SCS) assesses perceptions about potential negative consequences of taking the medicine [26]. The BMQ-D has response categories on a five-point Likert scale (1 = strongly disagree to 5 = strongly agree).

The nonvalidated items on medication lists were deduced from previously conducted focus groups (*n* = 2), interviews with medical experts (*n* = 26), and patient interviews (*n* = 8). The response categories of these items were partly dichotomous (yes/no) and partly scaled on a five-point Likert scale (0 = never to 4 = always).

### 2.3. Statistical Analysis

All statistical analyses were performed with SPSS, version 21.0 for Windows. For the SNS and SCS the mean, standard deviation and 95% confidence interval of the items belonging to the respective scale were calculated, resulting in a score ranging from one to five, higher values indicating stronger concerns or stronger perception of necessity, respectively. One item of the SCS was invalid due to a mistake in the wording. For this scale the mean of all valid items was calculated. A missing value was set if a participant had one or more missing values for any of the included statements.

The correlation between each BMQ-D subscale and the background variables was calculated using Spearman's correlation coefficient or Pearson correlation coefficient, respectively. Variables with sufficient potential interest (*p* < 0.20) as well as the sociodemographic data were included into two linear regression models and handled as independent variables. The linear regression analyses were carried out for the two subscales of BMQ-D, which were treated as dependent variables. An alpha level of *p* < 0.05 was used for statistical significance.

## 3. Results

### 3.1. Characteristics of the Sample

Table 1 shows the characteristics of the sample. In total, 344 patients completed the survey. The average age was 72.1 years, and 58% (*n* = 198) of the participants were female. The majority of patients were not working any more (85%, *n* = 293), living in a multiperson household (69%, *n* = 238), and having a long-term relationship (66%, *n* = 226).

The descriptive results of BMQ-D scales, medication lists, and medication counselling are depicted in Table 2.

### 3.2. Patients' Beliefs in Medicine

The percentage of missing values for the BMQ-D SNS was 0% and for the BMQ-D SCS 2.3%. The mean score for the BMQ-D SNS was 4.34 on a scale from 1 (strongly disagree) to 5 (strongly agree), reflecting a general strong belief in the necessity of the medication actually taken by the patients, whereas the mean score for the BMQ-D SCS was 2.47 on a scale from 1 (strongly disagree) to 5 (strongly agree), reflecting moderate concerns towards the prescribed medication.

### 3.3. Patients' Use of Their Medication List

As Table 2 shows, on average patients stated finding the information on their medication list frequently or always comprehensible (mean = 3.52) and receiving a new medication list after their medication was altered frequently or always (mean = 3.63). Scores were lower for the items referring to the active use of the medication list by the patients. They stated carrying their medication list rarely to sometimes with them (mean = 1.64) and updating their medication list never to rarely (mean = 0.77) when buying an over-the-counter drug. About half of the patients (50.6%, *n* = 174) considered their medication list an important reminder and 40% (*n* = 146) used it as aid when administering their medication. About one-third (30.2%, *n* = 104) stated showing their medication lists during doctor's appointments, but only a minority (4.1%, *n* = 14) did so when buying a drug in the pharmacy.

### 3.4. Patients' Memory of Medication Counselling and Brown Bag Review

About two-third of the patients (62.8%, *n* = 216) remembered an appointment for medication counselling at their GP, but only one-third stated having brought their medication packages to this appointment (thus to have received a “brown bag review”) as recommended.

### 3.5. Association between Memory of Medication Counselling, Use of Mediation Lists, and Beliefs in Medicine

Tables 3 and 4 show the results of the linear regression models related to the BMQ-D SNS or BMQ-D SCS, respectively, both controlled for sociodemographic data. The items “I usually show my medication list during doctor's appointments,” “I usually show my medication list when buying a drug in the pharmacy,” and “I usually use my medication list when taking my medication” were not included into the regression model of BMQ-D SNS since *p* > 0.20. Moreover, the items “Do you receive an updated medication list from your GP if your medication changes?” and “I usually show my medication list when buying a drug in the pharmacy” were not included into the regression model of BMQ-D SCS since *p* > 0.20.

Regular receipt of an updated medication list was associated with higher perceived necessity of the medication, while the memory of a “brown bag review” was negatively associated with perceived necessity. Patients who had stronger concerns towards their medication were more likely to remember a “brown bag review,” to carry their medication list along and to update their medication list when buying over-the-counter drugs. Patients who found their medication list comprehensive had less concerns about their medication.

## 4. Discussion

In our study patients' memory of a brown bag review and the use of a medication list correlated with their beliefs about their medicine.

The memory of a brown bag review was associated with stronger concerns and lower perceived necessity about the medication. This seems to contradict with the general consensus that medication reviews are valuable instruments to increase medication safety [27]. The finding suggests that also potential negative psychological effects of intensive medication counselling should be considered. This is in line with the concerns of some doctors to unsettle patients by giving too detailed information about medicines, especially about possible side-effects, which we identified as potential barrier for the implementation of medication counselling in previous qualitative studies [28, 29].

On the other hand, stronger concerns were associated with more active patient behaviour. Patients who had stronger concerns about their medication were more likely to carry their medication list with them and to add over-the-counter drugs on the list. This contrasts with the general assumption that concerns have to be minimized in order to increase adherence [30] and supports the importance of addressing patients' attitudes and feelings towards their medication respecting differences in personalities. Minimising concerns leading to nonadherence among “anxious” patients might be just as important as raising awareness for possible risks of pharmacotherapy among “careless” patients.

Finding this balance might be a challenge for health care professionals and require special pharmacological knowledge and conversational skills. In fact, there is little guidance on what level of detailed medication counselling should be conducted. Checklists for medication counselling usually specify general conversation topics [20, 22] but do not concretise the essential information to be given about different types of drugs. Further research should focus on methods to train and guide doctors and nurses in medication counselling and brown bag reviewing. Quality standards for these important care processes should be developed, for example, by elaborating the essential information that must be conveyed and collected during medication counselling on the level of the active ingredients of a medication.

In our study, patients who found their medication list most comprehensive had less concerns towards their medication and regular receipt of an updated medication list was associated with higher perceived necessity of the medication. This underlines the importance of establishing a standardized, high-quality medication list and also the need of empowering patients in the use of it. Therefore we argue that instructions on how to use medication lists correctly should be part of medication counselling and included into respective checklists.

This study has some strengths and limitations, which should be considered when interpreting results. Beside self-developed questions we used internationally validated measures for the evaluation of patient beliefs on medicines. However, our sample may not be representative for all patients with multiple chronic conditions and polypharmacy in Germany, although the age and gender patterns are comparable to those of a large German cohort study on multimorbid patients [31]. Moreover, this was an exploratory study; *p* values should be interpreted only in an explorative manner and need to be confirmed in further targeted studies.

## 5. Conclusions

The results of our study indicate that regular receipt of an updated and comprehensive medication list may reduce patients' concerns and increase the perceived necessity of their medication. This supports the demand to establish standardized, high-quality medication lists and to instruct patients in using them. Our findings suggest as well that potential negative effects of intensive medication counselling on patients beliefs about their medicine should be taken into consideration. Consequently, quality standards for the course and contents of structured medication counselling, ideally on the level of active agents, should be developed.

## Figures and Tables

**Table 1:** Characteristics of the survey respondents (*n* = 344).

Characteristics	
Age in years; mean (SD) (range)	72.1 (SD 8.94) (52–94)
Female, % (*n*)	57.6 (198)
Having a long-term relationship, % (*n*)	65.7 (226)
Living with other persons, % (*n*)	69.2 (238)
Not working, % (*n*)	85.2 (293)
High school or university degree, % (*n*)	4.9 (17)
Secondary modern school qualification, % (*n*)	76.7 (264)

**Table 2:** Descriptive results of BMQ-D, medication list, and medication counselling.

Beliefs about medicine^**∗**^	Mean	SD	95% CI
BMQ-D “specific-necessity scale”	4.34	0.59	4.29–4.41
BMQ-D “specific-concerns scale”	2.47	0.89	2.37–2.57

Use of the medication list^*∗∗*^	Mean	SD	CI

Do you find the information on your medication list comprehensive?	3.63	0.65	3.56–3.71
Do you receive an updated medication list from your GP when your medication changes?	3.52	0.86	3.42–3.62
Do you discard your previous medication list after receiving a new one?	3.11	1.33	2.96–3.26
Do you carry your medication list with you (e.g., in your purse)?	1.64	1.65	1.45–1.83
Do you note down on your medication list when you have a bought a new drug?	0.77	1.31	0.62–0.92

Use of medication list	Yes % (*n*)		

My medication list is an important reminder for me.	50.6 (174)		
I usually show my medication list during doctor's appointments.	30.2 (104)		
I usually show my medication list when buying a drug in the pharmacy.	4.1 (14)		
I usually use my medication list when taking my medication.	42.4 (146)		

Memory of medication counselling and “brown bag review”	yes % (*n*)		

Have you ever received “medication counselling” (an appointment, during which you explicitly talked about your medication) by your general practitioner?	62.8 (216)		
If yes, did you bring all medication packages, you are using, to this appointment (so called “brown bag review”)?	32.0 (110)		

^*∗*^Beliefs about Medicines Questionnaire (BMQ): scores possibly range from 1 to 5, higher values indicating higher perceived necessity or concerns, respectively. ^*∗∗*^Items assessing the use of medication lists: scores possibly range from 0 to 4 (0 = never, 4 = always).

**Table 3:** Associations of individual characteristics, medication list, and medication counselling on BMQ-D “specific-necessity scale.”

	*β* (*p* value)
Do you receive an updated medication list from your GP if your medication changes?	0.286 (<0.01)
If yes, did you bring all medication packages, you are using, to this appointment (so called “brown bag review”)?	−0.268 (0.01)
*R* ^2^	**0.152**

Results of stepwise linear regression analysis, under specification of standardized beta coefficient, *α* = 5%. Only the last step and coefficients with statistically significances at *p* < 0.05 level are reported.

**Table 4:** Associations of individual characteristics, medication list, and medication counselling on BMQ-D “specific-concerns scale.”

	*β* (*p* value)
Do you carry your medication list with you (e.g., in your purse)?	0.224 (<0.01)
Do you find the information on your medication list comprehensive?	−0.224 (<0.01)
If yes, did you bring all medication packages, you are using, to this appointment (so called “brown bag review”)?	0.160 (0.02)
Do you note down on your medication list if you have a bought a new drug?	0.156 (0.032)
*R* ^2^	**0.140**

Results of stepwise linear regression analysis, under specification of standardized beta coefficient, *α* = 5%. Only the last step and coefficients with statistically significances at *p* < 0.05 level are reported.
